# Is active surveillance a good treatment option for stage 1 seminoma in a developing nation? Long-term outcomes from the Indian subcontinent

**DOI:** 10.3332/ecancer.2025.1994

**Published:** 2025-09-23

**Authors:** Aditya Dhanawat, Debdeep Samaddar, Bhagyashri Jadhav, Atul Tiwari, Kunal Jobanputra, Arnav Tongaonkar, Minit Jalan Shah, Nandini Menon, Priyamvada Maitre, Mahendra Pal, Amandeep Arora, Aparna Ringe, Archi Agrawal, Santosh Menon, Gagan Prakash, Vedang Murthy, Vanita Noronha, Kumar Prabhash, Amit Joshi

**Affiliations:** 1 Medical Oncology, Advanced Centre for Treatment, Research and Education in Cancer (ACTREC) and Tata Memorial Hospital (TMH), Homi Bhabha National Institute, Mumbai 400094, India; 2 Radiation Oncology, Advanced Centre for Treatment, Research and Education in Cancer (ACTREC) and Tata Memorial Hospital (TMH), Homi Bhabha National Institute, Mumbai 400094, India; 3 Surgical Oncology, Advanced Centre for Treatment, Research and Education in Cancer (ACTREC) and Tata Memorial Hospital (TMH), Homi Bhabha National Institute, Mumbai 400094, India; 4 Radio-diagnosis, Advanced Centre for Treatment, Research and Education in Cancer (ACTREC) and Tata Memorial Hospital (TMH), Homi Bhabha National Institute, Mumbai 400094, India; 5 Nuclear Medicine, Advanced Centre for Treatment, Research and Education in Cancer (ACTREC) and Tata Memorial Hospital (TMH), Homi Bhabha National Institute, Mumbai 400094, India; 6 Surgical Pathology, Advanced Centre for Treatment, Research and Education in Cancer (ACTREC) and Tata Memorial Hospital (TMH), Homi Bhabha National Institute, Mumbai 400094, India

**Keywords:** seminoma, active surveillance, chemotherapy, testicular cancer, India

## Abstract

**Background:**

Stage 1 seminoma is treated with high inguinal orchiectomy (HIO) followed by either chemotherapy, radiation therapy (RT) or active surveillance (AS).

**Methods:**

This was a retrospective analysis of a prospectively collected dataset of patients with seminoma treated at a comprehensive cancer care centre in India. Adolescent and adult males with stage 1 seminoma were included.

**Results:**

A total of 114 patients were analysed. The median age was 39 years (IQR: 32–48 years). Stage IA was more frequently seen and 105 (92.1%) patients underwent unilateral HIO. Chemotherapy was offered to 66 (57.9%) patients. AS was offered to 32 (28.1%) patients while RT was offered to 16 (14%). Only 14 (43.8%) of the 32 patients on AS strictly adhered to the institutional follow-up guidelines for at least 2 years post treatment. Of the 114 patients, 9 (7.9%) patients had radiological relapse, while 4 (3.5%) of them were symptomatic at relapse. Bleomycin, etoposide and cisplatin were the most common regimen offered on relapse. The median follow-up of the cohort was 70.6 months (95% CI: 59.1–82 months). The mean relapse-free survival (RFS) was 107.7 months (95% CI: 102.5–112.8 months). The 1-, 2- and 5-year RFS were 97.3%, 95.5% and 92.4%, respectively. The mean overall survival (OS) was 114.9 months (95% CI: 113.2–116.6 months). The 2-, 5- and 8-year OS were 100%, 98.9% and 98.9%, respectively. There was no statistically significant benefit of 2 cycles over 1 cycle of carboplatin in terms of median RFS (96.5 versus 108.8 months, *p* = 0.260) or 5-year OS (95% versus 100%, *p* = 0.192). There was no statistically significant difference in RFS (*p* = 0.355) or OS (*p* = 0.684) based on treatment offered at baseline. There was no difference in survival between patients who strictly adhered to follow-up guidelines versus those who did not.

**Conclusion:**

In a developing nation with constrained resources, AS remains a good treatment option for stage 1 seminoma with excellent long-term outcomes and freedom from the toxicities of chemotherapy.

## Background

Testicular cancer is rare globally, with an annual incidence of 72,040 cases worldwide [1]. The incidence rates of testicular cancer are highest in Norway (11.8 per 100,000 men) and lowest in India (0.5 per 100,000 men) and Thailand (0.4 per 100,000) [2]. Germ cell tumours (GCTs) comprise of 95% of testicular cancers and the ratio of seminoma to non-seminomatous germ cell tumours is roughly 1:1. Approximately 80% patients with seminoma initially present with stage 1 disease globally. However, in a resource-constrained setting, the patients often ignore the symptoms and present late to the healthcare owing to the indolent nature of the disease, lack of health education and poverty.

A radical high inguinal orchiectomy (HIO) should be performed to confirm the histological diagnosis of seminoma and provide local tumour control. Clinical stage 1 seminoma is limited to the testicle. Post HIO, these patients are offered chemotherapy, radiation therapy (RT) or active surveillance (AS) [3]. Early studies on long-term outcomes of adjuvant radiation in stage 1 seminoma showed 10-year survival >90% albeit with a higher risk of second cancer or cardiovascular diseases [4, 5]. Subsequent studies showed AS as a safe and effective option [6, 7]. Randomised data from the UK showed non-inferiority of adjuvant carboplatin to radiotherapy with fewer second cancers, lesser lethargy and time taken off work [8]. The disease-specific survival rate was 100% in a prospective study of 725 patients of clinical stage 1 seminoma from Germany, while the disease-free survival (DFS) was 92% for stage 1 in a retrospective study on 67 cases of testicular seminoma from Trivandrum, India [9, 10]. The relapse-free survival (RFS) was 83.1% in a retrospective study from Kolkata, India, which included 61 patients with stage 1 seminoma [11]. The DFS was 92.6% in 81 patients with stage 1 seminoma who were either offered RT or AS, which was previously published from our institute in the early 1990s [12]. In the era of chemotherapy with carboplatin as an effective treatment option and paucity of data on long-term outcomes in stage 1 testicular seminoma from India, we aim to conduct this retrospective analysis.

## Methods

### Study design

This was a retrospective analysis of a prospectively collected dataset of patients who attended the male genitourinary oncology clinic at a tertiary care comprehensive cancer centre in India from January 2014 to December 2022.

### Study participants

Male patients aged 15 years and above with a histological diagnosis of seminoma and clinical stage 1 were included. Patients who were not evaluated at our centre or previously treated outside were excluded.

### End points

The primary end point of this study was to determine the RFS of patients with stage 1 seminoma on different modalities of treatment. Our secondary end point was to determine the overall survival (OS) of patients with stage 1 seminoma.

### Study methodology

Details regarding the demographic information, disease stage, orchiectomy, treatment at baseline and treatment at relapse were documented. All investigations and management were performed by the treating oncologists at the male genitourinary oncology clinic after discussion in the multi-modality joint clinic in our hospital comprising of surgical, radiation and medical oncologists. The clinical staging of seminoma was done by contrast-enhanced computed tomography (CECT) scan of the thorax, abdomen and pelvis. Clinical stage 1 seminoma at our centre were offered orchiectomy (if not done previously), followed by 1 cycle of single agent carboplatin area under curve (AUC) 7 mg/mL/min or RT to a dose of 20 Gy in 10 fractions to para-aortic lymph nodes or AS. The follow-up protocol for AS comprised of clinical examination every 3 months for the first year, followed by every 6 months for the second year, and annually thereafter till 5 years. A CECT of the abdomen and pelvis was also performed every 6 months for 2 years and annually thereafter till 5 years. Patients who defaulted on atleast one scheduled clinic visit or CECT scan were considered as non-adherent. The follow-up protocol for those who received either chemotherapy or RT comprised of clinical examination every 6 months for 2 years and annually thereafter, along with annual CECT of the abdomen and pelvis till 3 years post-treatment. Disease relapse was documented based on CECT scan of the thorax, abdomen and pelvis.

### Statistical analysis

As this was a retrospective study, no formal sample size calculation was performed. We included all patients who fulfilled the study eligibility criteria in the time period of the study. Data analysis was performed in the Statistical Program for the Social Sciences (IBM Corp. Released 2017. IBM SPSS Statistics for Windows, Version 25.0. Armonk, NY: IBM Corp.) and R Studio (Version 1.4.1106; RStudio Team, 2021). We utilised R (Version 4.1.0) for all statistical computations and data visualisations. Descriptive statistics were presented using absolute numbers and simple percentages. Quantitative data were presented using median and interquartile range. The RFS was calculated from the date of diagnosis to the date of relapse and OS was from the date of diagnosis to the date of death. Post 2 years of primary therapy, patients who attended the out-patient clinic in the last 12 months were assumed to be alive. Kaplan–Meier method was used for estimation of median RFS and OS. Comparison of survival between different treatment options was calculated using the log-rank method.

## Results

A total of 132 patients of stage 1 seminoma visited our outpatient clinic within the study period, of which 114 patients were included and analysed. The median age was 39 years (IQR: 32–48 years) (Table 1). Stage IA was more frequently seen and 105 (92.1%) patients underwent unilateral HIO. Chemotherapy (single agent carboplatin) was offered to 66 (57.9%) patients, which was well tolerated. AS was offered to 32 (28.1%) patients and RT to 16 (14%). Patients with high-risk features, such as involvement of rete testis or tumour size more than 4 cm, were offered 2 cycles of single-agent carboplatin.

Toxicities were seen in 22 (33.3%) of 66 patients who received carboplatin. Two (3%) patients had grade III neutropenia, while the rest were grade I–II toxicities, which were managed on an outpatient basis (Table 2). Of the 22 patients who had toxicities, 11 (50%) patients had received 2 cycles of carboplatin. Toxicities were not associated with more cycles of carboplatin (*p* = 0.612).

Rete testes involvement was seen in 2 (6.3%), 9 (56.3%), 13 (36.1%) and 13 (43.3%) patients who were offered AS, RT, 1 cycle carboplatin and 2 cycles carboplatin, respectively. Pathological tumour size >4 cm was seen in 15 (46.9%), 11 (68.8%), 28 (77.7%) and 25 (83.3%) patients who were offered AS, RT, 1 cycle carboplatin and 2 cycles carboplatin, respectively.

Out of 114 patients, 9 (7.9%) patients had radiological relapse, of which 4 (3.5%) were symptomatic. Of these nine patients, seven patients had received chemotherapy at baseline while two were on AS. Of the seven relapsed patients who had received chemotherapy at baseline, four had received two cycles carboplatin while three had received one cycle carboplatin. The median baseline pathological tumour size was 7 cm (range: 4.5–11 cm) in those who relapsed. All patients who relapsed had a baseline pathological tumour size >4.5 cm. Bleomycin, etoposide and cisplatin (BEP) was the most common regimen offered on relapse, followed by etoposide and cisplatin (EP) (Table 3). Only 14 (43.8%) of the 32 patients in AS had strictly adhered to the follow-up guidelines for atleast 2 years post treatment.

**Figure 1.**
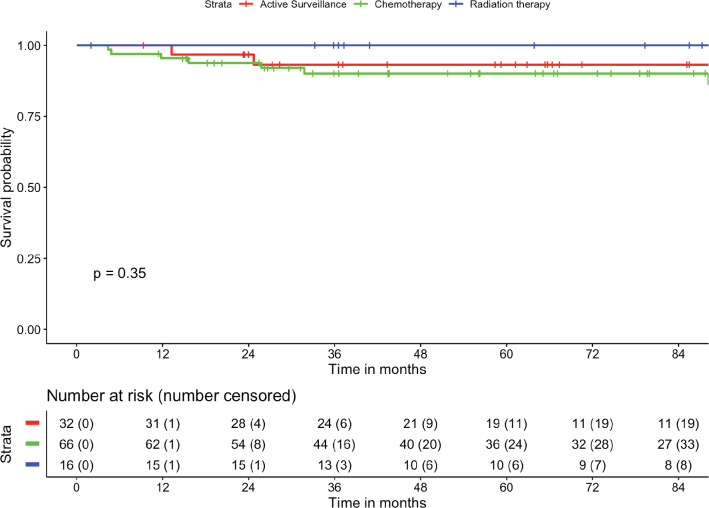
Estimation of RFS and stratified as per treatment modality.

**Figure 2.**
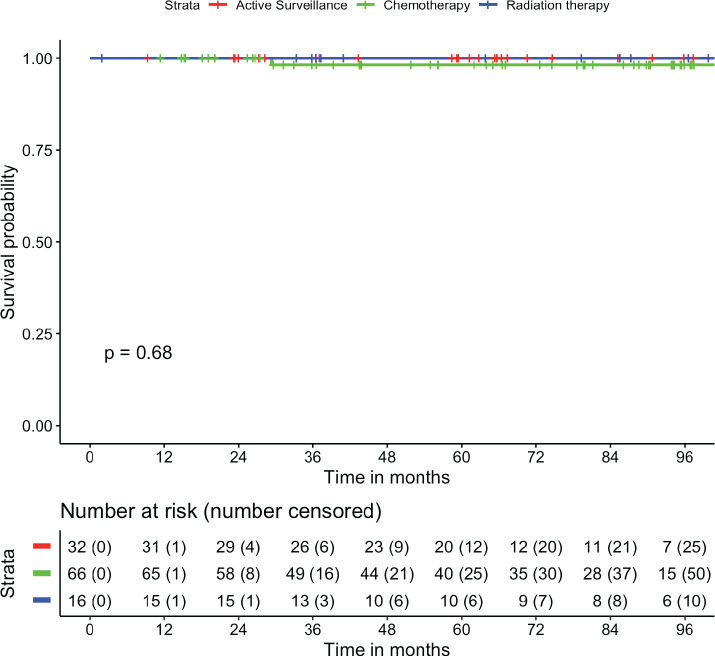
Estimation of OS and stratified as per treatment modality.

The median follow-up of the cohort was 70.6 months (95% CI: 59.1–82 months). There were no patients who were lost-to-follow up. The mean RFS was 107.7 months (95% CI: 102.5–112.8 months). The 1-, 2-, 5- and 7-year RFS were 97.3%, 95.5%, 92.4% and 92.4%, respectively (Figure 1). The mean OS was 114.9 months (95% CI: 113.2–116.6 months). The 2-, 5- and 8-year OS were 100%, 98.9% and 98.9%, respectively (Figure 2). There was no statistically significant difference in RFS (*p* = 0.355) or OS (*p* = 0.684) based on treatment offered at baseline. The mean RFS-2 (post salvage treatment) was 69.0 months (95% CI: 55.5–82.4 months).

## Discussion

The European Association of Urology guidelines on diagnosis and management of testicular cancers in 2023 recommended stage 1 seminoma could be treated with AS or adjuvant chemotherapy. Adjuvant RT should be reserved for highly selected patients not suitable for surveillance and with a contraindication for chemotherapy [13]. They also identified tumour size (>4 cm) and invasion of the rete testis as a predictor of relapse in stage 1 seminoma. In our study, all patients who relapsed had a tumour size >4.5 cm. Of the 9 relapsed patients, 5 (55.5%) had involvement of rete testis while 5 (55.5%) had elevated lactate dehydrogenase (LDH) post orchidectomy (range: 320–1,670 U/L) which has been recently iterated by International Germ Cell Cancer Collaborative Group and Global Germ Cell Tumour Collaborative Group (G3) as an independent risk factor [14, 15]. However, LDH alone does not help in early relapse detection in stage 1 seminoma [16, 17].

Prospective risk-adapted study from SWENOTECA included 897 patients of stage 1 seminoma who were offered surveillance (none or one risk factor) or one dose of carboplatin (AUC 7) (both risk factors). Among patients with one or both risk factors, 15.5% of the surveillance cohort experienced relapse versus 9% of the adjuvant carboplatin cohort [18]. Another study by the Spanish Germ Cell Cancer Group found a relapse rate of 11.1% and a 5-year DFS was 92.3%. Those who were on AS had a relapse rate of 14.8% whereas those on chemotherapy was 3.2% [19]. As per the MRC TE19/EORTC 30982 study, which had randomised 1,447 patients of stage 1 seminoma, the 5-year relapse-free rates were 94.7% (for carboplatin) and 96% (for RT), with a clear reduction in contralateral GCTs in patients who received carboplatin [20]. Our study showed an overall relapse rate of 7.9%, of which those on AS had a relapse rate of 6.25% while those on chemotherapy was 10.6%. The 5-year RFS of our patients was 95.5% which was comparable with previously published randomised evidence. Dabkara *et al* [11]

from Kolkata, India reported the 7-year OS of 98.7% which was comparable to our study, which showed an 8-year OS of 98.9%. A study by Anjanappa *et al* [21] from India reported poor compliance to AS in which 50% did not report after decision making. But in our study, even though 43% in AS strictly adhered to guidelines for 2 years, the remaining did continue to follow-up, although not strictly adhering to the date of scheduled follow-up.

Despite only 43.8% of patients on AS strictly adhering to follow-up guidelines for at-least 2 years, there was no difference in long-term outcomes as compared to chemotherapy or RT in our study. Moreover, 33.3% patients who received chemotherapy did have some toxicity. The median RFS-2 in our patients was 69 months, which indicates that salvage treatment is effective irrespective of the treatment chosen at baseline. Therefore, a more lenient follow-up may be practiced for patients on AS. This will reduce economic burden on the patients and possibly limit radiation exposure by reducing the frequency of scans in a developing nation.

This study represents the largest cohort of stage 1 seminoma from the Indian subcontinent. We have analysed the long-term RFS and OS in these patients. Our results reaffirm that AS, chemotherapy or RT are acceptable treatment options in stage 1 seminoma, while AS is a good treatment option that can be utilised in resource-constrained settings with less stringent follow-up guidelines. However, this is still a small cohort of 114 patients subject to bias and limited statistical power. AS was considered in only one-third of the patients since adherence to a strict follow-up schedule is not feasible at most of the time in developing countries due to financial and logistical reasons. A less stringent follow up can be considered in developing countries; however, this needs a randomised study to conclusively prove the hypothesis.

## Conclusion

In a developing nation with constrained resources, AS remains a good treatment option for stage 1 seminoma with excellent long-term outcomes and freedom from the toxicities of chemotherapy.

## Conflicts of interest

None.

## Funding

None.

## Figures and Tables

**Table 1.** Baseline characteristics.

Baseline characteristics	All patients (*n* = 114)
Age group	
Less than 30	22 (19.3%)
30–50	73 (64%)
More than 50	19 (16.7%)
Stage	
IA	73 (64%)
IB	41 (36%)
Orchiectomy	
High-inguinal	105 (92.1%)
Trans-scrotal	9 (7.9%)
Treatment	
AS	32 (28.1%)
Chemotherapy (Single agent carboplatin AUC-7)	66 (57.9%)
RT	16 (14%)
Number of cycles of chemotherapy	(*N* = 66)
One	36 (54.5%)
Two	30 (45.5%)

**Table 2.** Toxicities in patients who received carboplatin.

All patients who received carboplatin	(*N* = 66)
Grade I–II toxicities	20 (30.3%)
Fatigue	10 (15.2%)
Nausea/vomiting	9 (13.6%)
Thrombocytopenia	4 (6.1%)
Neutropenia	2 (3%)
Elevated liver enzymes	2 (3%)
Grade III–IV toxicities	2 (3%)
Neutropenia	2 (3%)

**Table 3.** Treatment details at relapse.

Stage at relapse	(*N* = 9)
Stage 1	1 (11.1%)
Stage 2	4 (44.4%)
Stage 3	4 (44.4%)
Modality of treatment at relapse	(*N* = 9)
Chemotherapy	8 (88.9%)
Surgery	1 (11.1%)
Chemotherapy at relapse	(*N* = 8)
BEP	4 (50%)
EP	2 (25%)
VIP (Etoposide, ifosfamide and cisplatin)	1 (12.5%)
PVB (Cisplatin, vinblastine and bleomycin)	1 (12.5%)

## References

[1] Sung H, Ferlay J, Siegel RL (2021). Global cancer statistics 2020: GLOBOCAN estimates of incidence and mortality worldwide for 36 cancers in 185 countries. CA Cancer J Clin.

[2] Curado M, Edwards B, Shin H (2007). Cancer Incidence in Five Continents.

[3] Albers P, Albrecht W, Algaba F (2015). Guidelines on testicular cancer: 2015 update. Eur Urol.

[4] Giacchetti S, Raoul Y, Wibault P (1993). Treatment of stage I testis seminoma by radiotherapy: long-term results - a 30-year experience. Int J Radiat Oncol Biol Phys.

[5] Zagars GK, Ballo MT, Lee AK (2004). Mortality after cure of testicular seminoma. J Clin Oncol.

[6] von der Maase H, Specht L, Jacobsen GK (1993). Surveillance following orchidectomy for stage I seminoma of the testis. Eur J Cancer.

[7] Warde P, Gospodarowicz MK, Panzarella T (1995). Stage I testicular seminoma: results of adjuvant irradiation and surveillance. J Clin Oncol.

[8] Oliver RT, Mason MD, Mead GM (2005). Radiotherapy versus single-dose carboplatin in adjuvant treatment of stage I seminoma: a randomised trial. Lancet.

[9] Dieckmann KP, Dralle-Filiz I, Matthies C (2016). Testicular seminoma clinical stage 1: treatment outcome on a routine care level. J Cancer Res Clin Oncol.

[10] James FV, Mathew A, Anand RK (2005). Testicular seminoma: review of 67 cases from India. J Clin Oncol.

[11] Dabkara D, Ganguly S, Ghosh J (2021). Clinicopathological characteristics, prognostic factors and treatment outcomes of seminomatous germ cell tumours from a tertiary cancer centre in eastern India. Natl Med J India.

[12] Tongaonkar HB, Dalal AV, Kelkar DS (1993). Stage I testicular tumours: the Tata Memorial Hospital experience. J Surg Oncol.

[13] Patrikidou A, Cazzaniga W, Berney D (2023). European association of urology guidelines on testicular cancer: 2023 update. Eur Urol.

[14] Gillessen S, Sauvé N, Collette L (2021). Predicting outcomes in men with metastatic nonseminomatous germ cell tumors (NSGCT): results from the IGCCCG update consortium. J Clin Oncol.

[15] Seidel C, Daugaard G, Nestler T (2021). The prognostic significance of lactate dehydrogenase levels in seminoma patients with advanced disease: an analysis by the Global Germ Cell Tumor Collaborative Group (G3). World J Urol.

[16] Bobrowski A, Anson-Cartwright L, Kuhathaas K (2022). Role of lactate dehydrogenase in identifying relapse for patients with stage I testicular cancer on surveillance. J Urol.

[17] Vesprini D, Chung P, Tolan S (2012). Utility of serum tumor markers during surveillance for stage I seminoma. Cancer.

[18] Tandstad T, Ståhl O, Dahl O (2016). Treatment of stage I seminoma, with one course of adjuvant carboplatin or surveillance, risk-adapted recommendations implementing patient autonomy: a report from the Swedish and Norwegian Testicular Cancer Group (SWENOTECA). Ann Oncol.

[19] Aparicio J, Maroto P, García Del Muro X (2014). Prognostic factors for relapse in stage I seminoma: a new nomogram derived from three consecutive, risk-adapted studies from the Spanish Germ Cell Cancer Group (SGCCG). Ann Oncol.

[20] Oliver RT, Mead GM, Rustin GJ (2011). Randomized trial of carboplatin versus radiotherapy for stage I seminoma: mature results on relapse and contralateral testis cancer rates in MRC TE19/EORTC 30982 study (ISRCTN27163214). J Clin Oncol.

[21] Anjanappa M, Kumar A, Mathews S (2017). Testicular seminoma: are clinical features and treatment outcomes any different in India?. Indian J Cancer.

